# Graph semantic similarity-based automatic assessment for programming exercises

**DOI:** 10.1038/s41598-024-61219-8

**Published:** 2024-05-08

**Authors:** Chengguan Xiang, Ying Wang, Qiyun Zhou, Zhen Yu

**Affiliations:** 1 Faculty of Artificial Intelligence in Education, Central China Normal University, Wuhan, 430079 China; 2 School of Mathematics and Big Data, Guizhou Education University, Guiyang, 550018 China

**Keywords:** Automatic assessment, Program dependency graph, Program semantics, Similarity, Programming exercises, Computer science, Software

## Abstract

This paper proposes an algorithm for the automatic assessment of programming exercises. The algorithm assigns assessment scores based on the program dependency graph structure and the program semantic similarity, but does not actually need to run the student’s program. By calculating the node similarity between the student’s program and the teacher’s reference programs in terms of structure and program semantics, a similarity matrix is generated and the optimal similarity node path of this matrix is identified. The proposed algorithm achieves improved computational efficiency, with a time complexity of $O(n^2)$ for a graph with n nodes. The experimental results show that the assessment algorithm proposed in this paper is more reliable and accurate than several comparison algorithms, and can be used for scoring programming exercises in C/C++, Java, Python, and other languages.

## Introduction

Programming is one of the basic skills required by students of computer-related majors in colleges and universities^1^. In the teaching of programming courses, the assessment of exercises submitted by students forms the basis for feedback that is intended to stimulate the students’ interest in learning. However, the manual assessment of students’ programming exercises is time-consuming, labor-intensive, and error-prone^2^. Replacing manual assessment with automatic assessment would not only reduce the workload of teachers, but also provide instant feedback through the assessment results, thereby enhancing the learning experience. There has been extensive research on the automatic assessment of program tasks, with the two main directions being dynamic assessment and static assessment^3,4^. The scoring mechanism used in dynamic assessment is based on the number of test cases passed by the student’s program. Many popular online judge systems use dynamic program assessment. However, this method can only assess executable code that runs without errors^4^. Student codes with a few wrong but almost-correct answers will be assigned zero points, which stifles the confidence of learners^5^ and leads to a significant decline in learning attitudes and motivation^6^, making it difficult for the students’ scores in the class to follow a normal distribution^7^. The scoring mechanism used in static assessment calculates the similarity between the student’s program and the teacher’s programs through statistical analysis, without executing the student code. In other words, the grading mechanism does not execute the student’s code to assess its correctness or performance, and it does not consider compilation errors or runtime errors. Instead, it analyzes the structure, syntax, semantics, and other characteristics of the student’s program, compares them with the teacher’s program, and calculates the similarity between them. This method simulates the teacher’s assessment process to a certain extent^8^ and is more suitable for the actual programming teaching environment^9^. It is evident that similarity-based static evaluation methods hold greater promise. Recently, these methods have primarily included those based on latent semantic analysis^10,11^, information retrieval^12^, syntax trees and program dependency graphs^13–15^, and machine learning^16–22^. While these methods have shown certain effectiveness in certain scenarios, they also face numerous challenges. These include issues such as the lack of training data due to the absence of student code for new problems, reliability issues stemming from the diversity of programming languages, and evaluation efficiency issues caused by low graph computation efficiency.

To address these challenges and enhance the accuracy and efficiency of evaluation. In this paper, a graph semantic similarity-based automatic assessment method for programming exercises is proposed. This method calculates the code similarity from the structural similarity of the program dependency graph and the semantic similarity of the nodes, and then assigns a score to the student’s code. The specific contributions of this paper are as follows:

1. Taking the structural similarity of the program dependency graph and the semantic similarity of the nodes as the assessment criteria, a new automatic assessment algorithm for student’s programming exercises is proposed.
2. By calculating the node similarity between the student’s program and the teacher’s programs, a similarity matrix is generated. An optimal similar node path matching algorithm is proposed, which improves the efficiency of calculating the similarity of the program dependency graph. For a program dependency graph with n nodes, the time complexity is $O(n^2)$.
3. The assessment algorithm proposed in this paper can be used to score programs that have compilation errors or cannot be executed correctly, and supports the scoring of programs in multiple programming languages.

The remainder of this paper is organized as follows. “Related work” outlines the relevant research work on automatic evaluation of program assignments. “Preliminaries” introduces some preliminaries required to develop the proposed algorithm. “Automatic assessment algorithm for program work based on graph semantic similarity” describes the proposed assessment algorithm in detail, before “Experiments” presents the results of an experimental verification process. Finally, “Conclusion” summarizes the results of this study.

## Related work

Since the 1960s, researchers have been continuously exploring and studying the technology of automatic code evaluation. In 1965, the first automated testing system for programming appeared, achieving a breakthrough from 0 to 1^23^.

From 1980 to 1999, automatic programming code evaluation technology emerged in the form of command-line or graphical user interface tools, requiring the design of a set of commands to execute student programs, focusing on evaluating whether the program is correct^24^. Hung et al.^25^ proposed an automatic scoring algorithm for programs based on software metrics, considering factors such as programming skills, complexity, programming style, and program efficiency.Reek^26^ developed the TRY system, which aims to provide accurate and objective evaluation methods, by using teachers’ test data to evaluate the running performance of student programs.Joy and Luck^27^ developed an online programming assignment submission and testing system called BOSS, which allows students to perform self-tests before submitting their programs to ensure their correctness.The Ceilidh automatic scoring system not only evaluates whether the program is correctly executed and whether the programming style is standardized, but also further analyzes the complexity of the program structure^28^.

From 2000 to 2010, the rise of the Internet has greatly influenced the way people work and learn, and web-based software architectures have become the new trend of the era. Programming code automatic evaluation has also ushered in a golden age of development^24,29,30^. Jackson^31^ argues that the challenge in automatic assessment of student programming assignments lies in ensuring that the program output is entirely correct, and proposes that a combination of human and machine evaluation can better handle assignment assessment.Truong et al.^32^ introduced a static analysis framework that utilizes software engineering metrics to assess program quality. They selected a method based on the XML representation of program abstract syntax trees to analyze and validate the structural similarity of student solutions. Romli et al.^33^ argue that in evaluations, the focus should not only be on detecting program errors, but also on assessing programming assignments through program static analysis to ensure effective achievement of teaching objectives.

Since 2011, the rapid development of information technologies such as big data, cloud computing, the Internet of Things, and the Internet has promoted the rapid development of artificial intelligence. Integrating artificial intelligence techniques into similarity-based static evaluation of programming code has become one of the focal points of researchers’ attention and has yielded significant research achievements in this direction. Zen et al.^10^ proposed an algorithm for automatically scoring programming exercises using latent semantic analysis(LSA), with a semantic vector space constructed by extracting the code structure and the cosine similarity between the student’s exercises and answers calculated as the assessment criterion. Xu et al.^34^ propose a multi-granularity feature fusion automatic scoring method based on latent semantic analysis. They extract features from student programs and standard answer template programs, and calculate the similarity between the features. Inturi et al.^11^ proposed the Programming Assignment Grading through Control Statement and Program Features (PAGCSPF) algorithm. This is a novel similarity measurement method that utilizes control statement features and program features to compare and compute the similarity between student program code and teacher program code based on their semantic execution patterns. The scoring results are similar to those of human teachers. Rahaman et al.^12^ proposed an evaluation model that automatically evaluates C programming assignments using the TF-IDF(Term Frequency-Inverse Document Frequency) algorithm. By constructing TF-IDF score vectors of student programs and teacher programs, the cosine similarity between the vectors is calculated to give a score. However, due to the richness of programming languages, such as the use of different identifiers for variables, the reliability of evaluation is poor. Verma et al.^13^ proposed the Syntax Tree Fingerprinting for Automated Evaluation(STF) grading algorithm, which calculates the similarity of syntax tree structures by extracting code fingerprint features, and verified the feasibility of this algorithm on codes written in Python. However, the algorithm only assesses the compiled code, ignoring the rich feature information in the program dependency graph, which directly affects the assessment accuracy. AlShamsi et al.^35^ proposed the Grader system for programming course evaluation, which uses program graph representation to assess structural similarity and software metrics to evaluate program quality. The system matches the output of student programs with the model recognition process to effectively evaluate Java source code. Zougari et al.^14^ converted the program into a control flow graph to calculate the graph similarity. However, when the number of nodes in the graph is large, the result cannot be accurately calculated in a reasonable time^15^, resulting in low assessment efficiency. Additionally, researchers have employed models like ridge regression, random forest, convolutional neural networks (CNNs), long short-term memory (LSTM), among others, to analyze the code submitted by students. These models are used to compare the submitted code with the reference answer, automatically determining its correctness and quality^16^. Srikant et al.^17^ achieved better scoring results using ridge regression models than scoring based on test cases. Lazar et al.^18^ used random forest in automatic program evaluation algorithm, achieving a correct rate of over 85%. Rezende Souza et al.^19^ trained a convolutional neural network on collected exercises and achieved an average accuracy of 74.9%. Nabil et al.^20^ introduced long-term and short-term memory into the evaluation system to perform code analysis to detect syntax errors. Srikant et al .^21^ used machine learning to assess the similarity of student codes through the latent semantic modeling of grammatical features and code structures. Muddaluru et al.^22^ proposed a deep learning and statistics combination method to predict C programming code scores. The model was used for word vector conversion preprocessing, and CNN, random forest, and LSTM were integrated to predict the scores of programming assignments. However, in programming teaching, if teachers often use past questions to test students, it may lead to serious plagiarism by students. To avoid this situation, teachers need to use new questions to test students, to ensure that they can truly master programming skills and solve problems independently. However, new questions have fewer solutions, making it difficult to meet the data requirements for machine learning training. Therefore, it can be seen that there are defects in the practical programming teaching environment based on machine learning evaluation methods.

## Preliminaries

### Graph similarity calculation

Calculating the graph similarity is a key issue in the field of graph research, and is the basis for many downstream tasks. The graph similarity calculation is often based on graph kernel and graph matching methods. The graph kernel method first decomposes the graph into a combination of subgraph structures, and then measures the graph similarity by comparing the subgraph distributions of the two graphs. For graphs with n vertices, the time complexity of the classical Graphlet kernel method is $O(n^4)$^36^. In contrast, graph matching methods first calculate a certain similarity measure between two graphs through the cross-graph mechanism, such as the classical graph edit distance (GED) algorithm. Based on the greedy assignment problem, GED has a time complexity of $O((n+m)^2)$ when the two graphs contain n and m vertices, respectively^36,37^. However, the current core method of calculating the graph similarity is an NP-complete problem^15,38,39^. When the number of nodes in the two graphs is greater than 16, even the most advanced GED algorithm cannot accurately calculate the results within a reasonable time^15^.

### Relative entropy

The relative entropy is a measure of the difference between two independent probability distributions^40^, also known as the Kullback-Leibler (KL) divergence. For the same random variable *X*, if there are two independent probability distributions *P*(*x*) and *Q*(*x*), then the relative entropy *DKL*(*P*||*Q*) is defined as

1 $$\begin{aligned} D_{K L}(P \Vert Q)=\sum _{x \in X} P(x) \log \frac{P(x)}{Q(x)} \end{aligned}$$

### Maximum common subsequence

A sequence *c* that is a subsequence of both string *X* and string *Y* is called a common subsequence of *X* and *Y*. All common subsequences of *X* and *Y* constitute the set $C=\{c_1,c_2,\cdots ,c_n\}$. If $c_i(1\leqslant i \leqslant n)$ is the element with the largest length in the set, then $c_i$ is the largest common subsequence (LCS) of *X* and *Y*.

## Automatic assessment algorithm for program work based on graph semantic similarity

**Figure 1:**
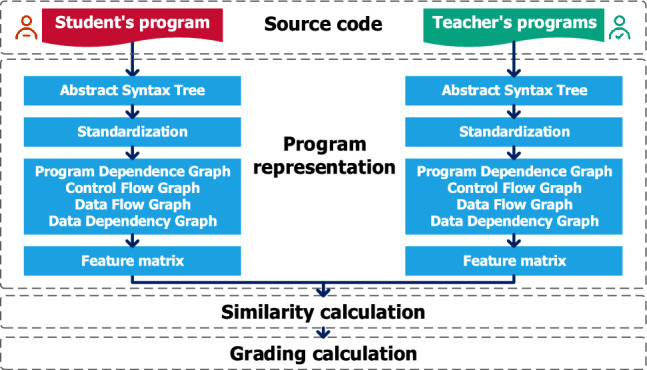
Algorithm flow of automatic assessment of program work based on the similarity of graph semantics.

**Figure 2:**
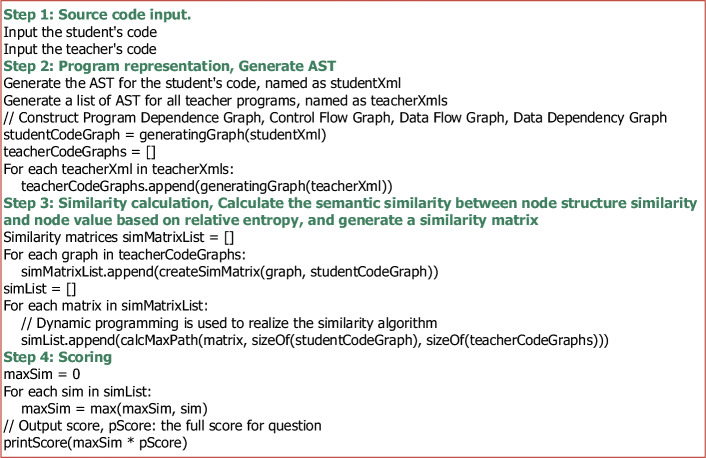
Pseudo-code representation of automatic assessment of program work based on the similarity of graph semantics.

In the process of teaching programming, when reviewing the program codes submitted by students, teachers often compare the similarity between the student’s program and the reference answers (search score points), and assign scores according to the degree of matching. Therefore, by calculating the similarity between the student’s program code and the correct codes given by the teacher, assessment methods based on the similarity score are closer to the characteristics of human assessment behavior^41^. This paper proposes a graph semantic similarity-based automatic assessment method for programming exercises, which solves the scoring problem in the assessment of students’ programming courses. The process of the proposed algorithm, which is illustrated in Fig. 1, includes four main steps, and the pseudo-code of the algorithm is shown in Fig. 2.

In this section, we mainly introduce four steps of automatic evaluation of program tasks based on graph semantic similarity.

Step 1: Source code input. The input is a collection of source code from the student’s program and the teacher’s programs. The student’s program source code refers to a single program text submitted by a student. The teacher’s programs source code refers to the collection of program source code giving various solutions corresponding to the problem.

Step 2: Program representation.

1. Generate abstract syntax tree
  Abstract syntax tree(AST) is a tree diagram representing the syntactic structure of a program. Each node on the tree represents a structure in the source code, and is used for compiler optimization, code generation, static analysis, and other tasks. To ensure that the assessment algorithm supports a variety of programming languages and meets the needs of programming teaching to the greatest extent, it is necessary to convert the source code of the student’s program and the teacher’s programs into a syntax tree. The syntax trees created by srcML (https://www.srcml.org) allow for convenient analysis and manipulation of the source code, with the tree structure represented by formatted xml. Thus, srcML is a lightweight, highly extensible, and robust multi-language syntax tree generation tool. The xml documents can be accessed using the jsoup tool (https://jsoup.org). For the simple case of three integer variables a, b, c in the C language declaration statement, the syntax tree representation generated by srcML is shown in Fig. 3.

**Figure 3:**
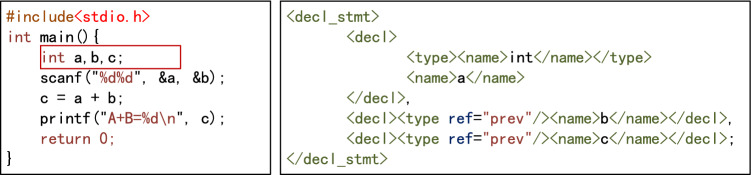
Example syntax tree generated by srcML.
2. Abstract syntax tree standardization
  The purpose of standardization is to eliminate syntactic differences between programs as far as possible. This reduces the number of teacher’s programs and improves the accuracy of assessment^3^.
  1. Variable declaration node standardization. The richness of programming languages means that variables can be declared in various forms. It is possible to declare only one variable after a data type identifier, or to declare multiple variables at the same time and initialize their values when declaring variables, such as int a,b,c = 0. In languages such as C++, Java, and Python, it is not necessary for all declaration statements to be placed at the beginning of the program. The standardization of syntax trees is intended to eliminate this variety. During standardization, only one variable can be declared after a data type identifier. A declaration containing multiple variables is decomposed into multiple statements, and the initialization assignment is decomposed into independent assignment statements. The declaration of all variables must be placed at the beginning of the largest program block in its scope. The variables are listed in descending order according to the number of references in the subsequent program (if the number of references is the same, they are listed in the order of the references). For example, the variable declaration statement int a = 1, b, c; in the C language can be standardized as int a; int b; int c; a=1;. The standardized syntax tree is shown in Fig. 4.
  2. Expression node equivalence standardization. Expressions that are semantically equivalent to each other have various forms of expression. The equivalence standardization of expression nodes is intended to eliminate the changes and differences between expressions, resulting in as unified a form as possible. All /=, %=, *=, −=, +=, ++, –, and other statements in the program are converted into general forms, e.g., $a/=b$ becomes $a=a+b$. Compound statement nodes are decomposed into multiple simple statement nodes, i.e., $x=y=z$ is decomposed into two nodes, $x=y$ and $y=z$. Symbolic calculation is used to simplify algebraic expressions and logical expressions. For example, the algebraic expression $y=x+3+3*x+y*0+x+1$ is normalized to $y=5*x+4$, and the logical expression $y=(a||a \&  \& b)$ is simplified to $y=a$.
  3. Node semantic standardization. In the programming process, students have considerable autonomy in the choice of identifiers such as variable names and function names, and it is impossible to force students to use uniform identifiers^10^. For example, with the statement for(int i=0;i<n;i=i+1), students could write for(int low=0;low<len; low=low+1). To eliminate this semantic difference, all identifiers are replaced with “#” in statements, and arithmetic operators, logical operators, parentheses, and constants remain unchanged. For example, the statement for(int i=0;i<n;i=i+1) can be mapped to the latent semantics #(# #=0 # < # # = # + 1), and the statement if(n>m && x<y) can be mapped to the latent semantics #(# > # && # < #). Finally, the value of the node is marked as a latent semantic string.
  4. Delete nodes with invalid semantics. Deleting all sentences that do not contain actual semantics will improve the accuracy of the assessment. Thus, combined with a previous suggestion^2^, sentence nodes with invalid semantics are deleted according to the following rules.
    A. Delete statement nodes that do not modify any variable data, such as the statements $x=x$ and $x+3$;
    B. If two adjacent assignment statement nodes are assigned to the same variable and will not cause other variable values to be lost, delete the former node, such as for the two assignment statement nodes $x=y$ and $x=z$, where the node $x=y$ is deleted;
    C. If the variable is not used after being assigned, delete the expression node;
    D. After executing the first three deletion rules, delete all variable declaration nodes that are not referenced in their scope.
  5. Node structure standardization. In the syntax tree generated in the previous step, the nodes are reconstructed according to the statement, and the statement information is decomposed into node attributes. Because the program is executed from top to bottom, it is vital to maintain the consistency of each node in the syntax tree and the program source code. Thus, the middle root traversal method of the tree is used to number (ID) the nodes in the syntax tree, except for the root node. Nodes other than the root node are assigned natural numbers starting from 0, and root node is assigned a value of $-1$ (this refers to the root node of the syntax tree generated by srcML). For example, consider a program to calculate the value of a+aa+aaa+...+aa...a (where the number of digits in the final addend is *n*), where the values of n and a are input from the keyboard. The source code of a C program submitted by a student is shown in Fig. 5, and the syntax tree after the node structure has been standardized is shown in Fig. 6.

**Figure 4:**
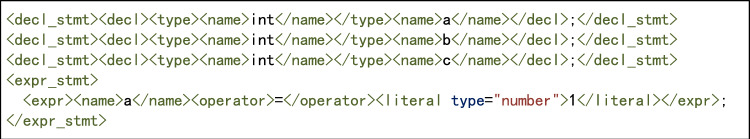
Example of a standardized syntax tree for variable declarations.

**Figure 5:**
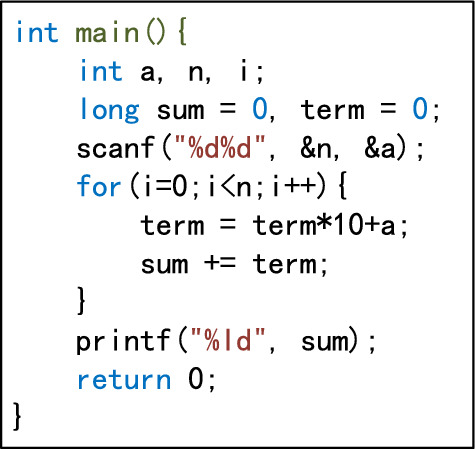
Program source code.

**Figure 6:**
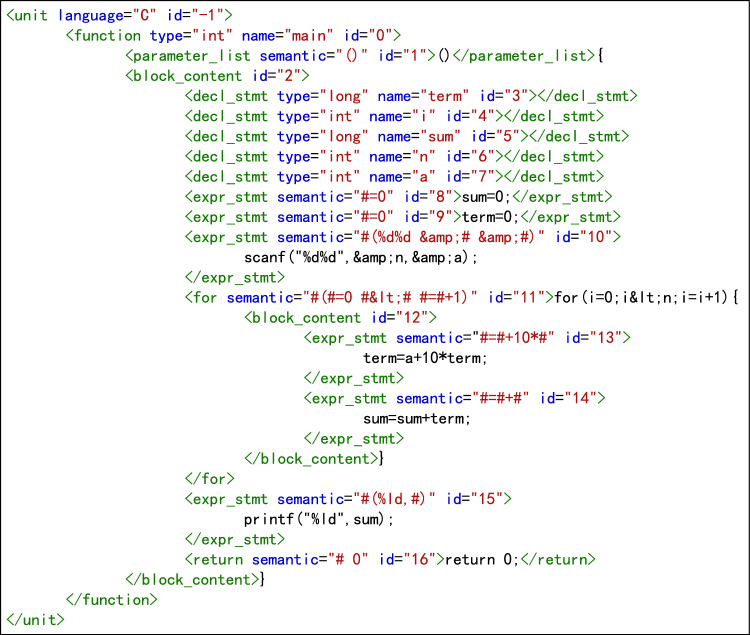
Normalized syntax tree.
3. Generating program dependency graph
  Program dependence graph(PDG) is a directed multigraph with labeled edges, used to represent the control and data dependencies in a program. The directed edges of the control flow graph(CFG), data flow graph(DFG), and data dependency graph(DDG) are then added to the standardized syntax tree to generate the program feature adjacency matrix. For the syntax tree shown in Figure 6, the generated program dependency graph is shown in Figure 7.

**Figure 7:**
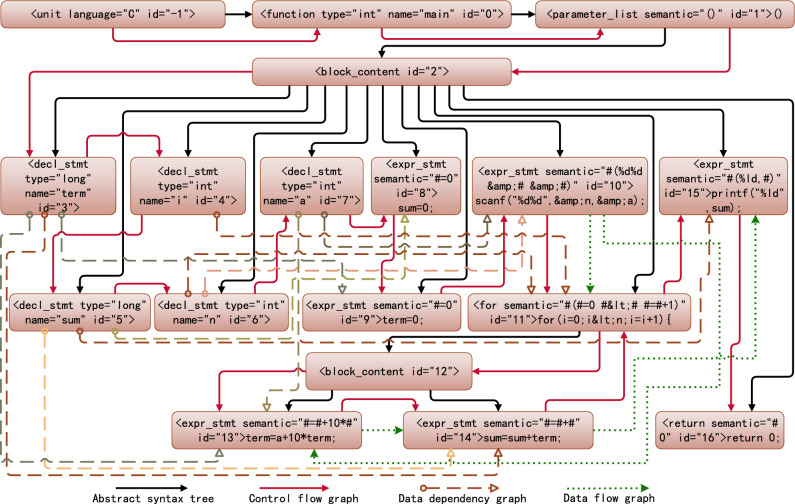
Normalized program dependency graph.
4. Generating graph adjacency matrix
  Neglecting the root node, which has an ID of $-1$, if the directed graph $A=(V, E)$ is the PDG, where *V* represents the set of *n* nodes and *E* represents the set of all directed edges, then the adjacency matrix can be represented as
  2 $$  \begin{aligned} M_{i \in V  \&   \&  j \in V}(i, j)=\left\{ \begin{array}{ll} 1 &{} \text{ if }<i, j>\in E \Vert <j, i>\in E \\ 0 &{} \text{ else } \end{array}\right. \end{aligned}$$

Step 3: Similarity calculation. After calculating the adjacency matrix of the dependency graph between the student’s program and the teacher’s template programs, the student’s program is matched with each of the teacher’s template programs and the relative entropy of structural information and semantic information is used to measure the similarity between nodes. This section describes a semantic similarity calculation method based on the relative entropy of the nodes and the maximum common subsequence.

**Figure 8:**
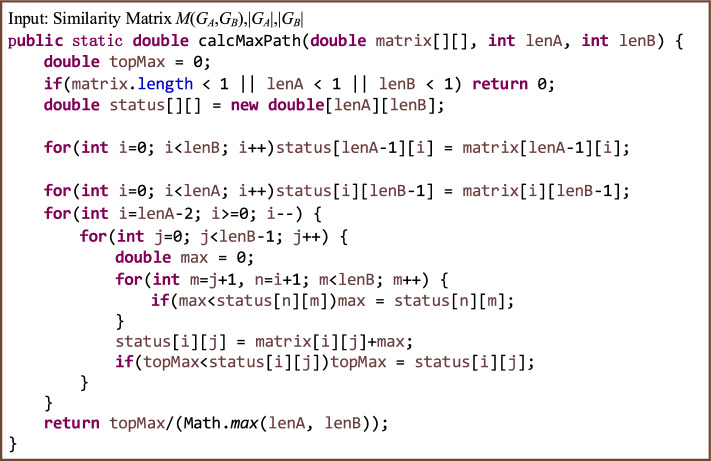
Dynamic programming for implementing graph similarity algorithm.

1. Calculate the similarity between each node in the dependency graphs of the student’s program and the teacher’s programs.
  Calculating the similarity between nodes is equivalent to finding the differences between local structural information. If the difference between nodes is relatively small, the similarity is relatively large, and vice versa^42^. Therefore, the relative entropy and semantics can be used to quantify the difference between nodes. The calculation method is divided into four steps: (a) Generate the probability set of nodes and adjacent nodes; (b) Calculate the relative entropy of the node pair; (c) Calculate the semantic maximum common subsequence length of all AST subtree nodes contained in the node pair; (d) Calculate the similarity of two nodes from the relative entropy and maximum common subsequence length.
  To clarify the calculation of the similarity between nodes, we use $G_A$ to represent the student’s program dependency graph and $G_B$ to represent the teacher’s programs dependency graph. Let nodes *x* and *y* be taken from the node sets of graphs $G_A$ and $G_B$, respectively, and let $|G_A|$ and $|G_B|$ represent the numbers of nodes in graphs $G_A$ and $G_B$.
  1. Generate the probability set of nodes and adjacent points A local network is composed of nodes and their adjacent points. Let $D(G_A)$ represent the degree of graph $G_A$ and $D(G_B)$ represent the degree of graph $G_B$, and $D_{max}=Max(D(G_A), D(G_B))$. The probability set of node *i*, *P*(*i*), is used to calculate the relative entropy, and node *i* itself is included in the calculation. Thus, *P*(*i*) should have $D_{max}+1$ elements, and is specifically expressed as follows:
    3 $$\begin{aligned} P(i)=\left[ p(i, 0), p(i, 1), \cdots , p\left( i, D_{\text{ max } }\right) \right] \end{aligned}$$
    When $D_{max}\geqslant k>0, p(i,k)$ represents the probability of the *k*th adjacent point of node *i*, and *p*(*i*, 0) represents the probability of node *i* itself, that is,
    4 $$  \begin{aligned} p(i, k)=\left\{ \begin{array}{ll} \frac{{\text {degree}}(i)}{{\text {degree}}(i)+\sum _{m=1}^{\min \left( {\text {degree}}(i), D_{\max }\right) } {\text {degree}}(m)} &{} k==0 \\ \frac{{\text {degree}}(k)}{{\text {degree}}(i)+\sum _{m=1}^{\min \left( {\text {degree}}(i), D_{\max }\right) } {\text {degree}}(m)} &{} k>0  \&   \&  k<={\text {degree}}(i) \\ 0 &{} k>{\text {degree}}(i) \end{array}\right. \end{aligned}$$
    For the PDG shown in Fig. 7, if the degree of the PDG it is being compared with is less than 12, then $D_{max}=12$, and the adjacent points of node 11 are 2, 4, 6, 10, 12, 14, and 15. Therefore, the probability set of node 11 is
    $$\begin{aligned} P(11)=\left[ \frac{7}{45}, \frac{12}{45}, \frac{4}{45}, \frac{5}{45}, \frac{5}{45}, \frac{3}{45}, \frac{5}{45}, \frac{4}{45}, 0,0,0,0,0\right] \end{aligned}$$
  2. Calculate the relative entropy of the node pair The relative entropy represents the asymmetric difference between two probability distributions. Both the relative entropy and similarity will be different when calculated using a different order of elements in the probability set^42^. Therefore, when calculating the relative entropy of a node pair, the first element of the probability set must be the probability of the current node, and the other elements are arranged in descending order of probability. Let $P'(i)$ represent the probability set of node *i* after sorting, which is expressed as follows:
    5 $$\begin{aligned} P^{\prime }(i)=\left[ p(i, 0), p^{\prime }(i, 1), \ldots , p^{\prime }\left( i, D_{\max }\right) \right] \end{aligned}$$
    For example, the probability set *P*(11) in the previous section is reordered as follows:
    $$\begin{aligned} P^{\prime }(11)=\left[ \frac{7}{45}, \frac{12}{45}, \frac{5}{45}, \frac{5}{45}, \frac{5}{45}, \frac{4}{45}, \frac{4}{45}, \frac{3}{45}, 0,0,0,0,0\right] \end{aligned}$$
    Let $P'(x)$ represent the probability set of nodes in graph $G_A$ and $P'(y)$ represent the probability set of nodes in graph $G_B$. The relative entropy of *x* and *y* is defined as follows:
    6 $$\begin{aligned} \textrm{KL}\left( P^{\prime }(x) \Vert P^{\prime }(y)\right) =\sum _{m=0}^{\min ( \text{ degree } (x) \text{,degree } (y))} p^{\prime }(x, m) \ln \frac{p^{\prime }(x, m)}{p^{\prime }(y, m)} \end{aligned}$$
  3. Calculate the maximum common subsequence length of all AST subtree node semantics contained in the node pair Use the first root access nodes *x* and *y* as the AST subtrees of the root nodes, respectively, and connect the semantic attribute values of the visited nodes as the semantic sequence of the nodes, denoted as $token\_sequences(x)$ and $token\_sequences(y)$. In the PDG shown in Fig. 7, $token\_sequences(11)=``\#(\#=0 \#<\# \#=\#+1) \#=\#+10 *\# \#=\#+\#''$. The dynamic programming algorithm is used to calculate the maximum common subsequence length of nodes *x* and *y*, that is,
    7 $$\begin{aligned} LCS\_len(x, y)=length(LCS(token\_sequences(x), token\_sequences(y))) \end{aligned}$$
  4. Calculate the similarity of two nodes from the relative entropy and maximum common subsequence length The similarity between nodes *x* and *y* is quantified using the relative entropy of node pairs and the longest common subsequence. The similarity based on the relative entropy of node pairs is defined as follows:
    8 $$\begin{aligned} {\text {Sim}}_{\textrm{KL}}(x, y)=1-\frac{\textrm{KL}\left( P^{\prime }(x) \Vert P^{\prime }(y)\right) +\textrm{KL}\left( P^{\prime }(y) \Vert P^{\prime }(x)\right) }{2} \end{aligned}$$
    The similarity of the LCS based on the node pair is defined as follows:
    9 $$\begin{aligned} {\text {Sim}}_{\textrm{LCS}}(x, y)=\frac{2 \times {\text {LCS}}\_{\text {len}}(x, y)}{length(token\_sequences(x))+ length(token\_sequences(y))} \end{aligned}$$
    The similarity between nodes x and y is then defined as:
    10 $$\begin{aligned} {\text {Sim}}(x, y)=a \times {\text {Sim}}_{\textrm{KL}}(x, y)+b \times {\text {Sim}}_{\textrm{LCS}}(x, y) \end{aligned}$$
    where *a* is the similarity weight based on the relative entropy of the node pair, *b* is the similarity weight based on the LCS of the node pair, and $a+b=1$. The optimal values of *a* and *b* are determined by least-squares estimation, $a=0.6416$, $b=0.3584$. We then calculate the similarity between each node in $G_A$ and each node in $G_B$, and generate the node similarity matrix between $G_A$ and $G_B$:
    $$\begin{aligned} M\left( G_{A}, G_{B}\right) =\left[ \begin{array}{cccc} {\text {Sim}}(0,0) &{} {\text {Sim}}(0,1) &{} \cdots &{} {\text {Sim}}\left( 0,\left| G_{B}\right| -1\right) \\ {\text {Sim}}(1,0) &{} {\text {Sim}}(1,1) &{} \cdots &{} {\text {Sim}}\left( 1,\left| G_{B}\right| -1\right) \\ \vdots &{} \vdots &{} \vdots &{} \vdots \\ {\text {Sim}}\left( \left| G_{A}\right| -1,0\right) &{} {\text {Sim}}\left( \left| G_{\textrm{A}}\right| -1,1\right) &{} \cdots &{} {\text {Sim}}\left( \left| G_{A}\right| -1,\left| G_{B}\right| -1\right) \end{array}\right] _{\left| G_{A}\right| \times \left| G_{B}\right| } \end{aligned}$$
2. Graph similarity calculation
  Program assessment needs to return results within a reasonable time. To ensure the efficiency of the program dependency graph similarity calculation, this paper proposes a new optimal similar node path matching algorithm. The basic idea of the algorithm design is as follows:
  1. When teachers review students’ programs, they usually compare them against reference programs, identify the statements (points) that are most likely to score, and assign points as appropriate. Finally, the students’ scores are aggregated according to all points.
  2. For novice programmers, incentives are more conducive to stimulating learning interest than penalties. Thus, the assessment algorithm should attempt to determine the optimal pair of similar nodes, with each node only selected once.
  3. Because the program code is executed from top to bottom, the selection of the optimal similarity node pair must be carried out in sequence according to the AST first root sequence. In generating the optimal similarity path, the optimal similarity node for the current node can only appear in the first root sequence after the first pair of similar nodes.
  4. The similarity of two PDGs is measured as the sum of the similarity of all node pairs on the optimal similarity path of the similarity matrix. In the similarity matrix $M(G_A, G_B)$, for any (*v*, *w*), $0\leqslant v< |G_A|, 0\leqslant w<|G_B|$, the optimal similar path is:
    11 $$\begin{aligned} {\text {SMP}}(v, w)=\left\{ \begin{array}{ll} (v, w) &{} \left( v=\left| G_{A}\right| -1\right) {\text {or}}\left( w=\left| G_{B}\right| -1\right) \\ (v, w) \bigcup \left\{ {\text {SMP}}\left( v+1, w^{\prime }\right) \mid w^{\prime }>w,\right. &{} \\ w^{\prime }<\left| G_{B}\right| , {\text {SMV}}\left( \textrm{v}+1, w^{\prime }\right)>\forall \{ &{} \text{ others } \\ \left. \left. {\text {SMV}}(v+1, \beta )\left| \beta \ne w^{\prime }, \beta >w, \beta <\right| G_{B} \mid \right\} \right\} &{} \end{array}\right. \end{aligned}$$
    where *SMV* is the sum of the similarity of all nodes on the optimal similar path, namely:
    12 $$\begin{aligned} {\text {SMV}}(v, w)=\sum _{(\alpha , \beta ) \in S M P(v, w)} {\text {Sim}}(\alpha , \beta ) \end{aligned}$$
    The largest optimal similarity path with *SMV* is taken as the optimal similarity path of the similarity matrix $M(G_A, G_B)$. The similarity between graphs $G_A$ and $G_B$ is:
    13 $$\begin{aligned} {\text {Sim}}_{\text{ graph } }\left( G_{A}, G_{B}\right) =\frac{{\text {Max}}_{\left( v \in G_{A}, w \in G_{B}\right) }(S M V(v, w))}{{\text {Max}}\left( \left| G_{A}\right| ,\left| G_{B}\right| \right) } \end{aligned}$$
    Dynamic programming is used to realize the similarity algorithm for $G_A$ and $G_B$, as shown in Fig. 8. The time complexity of the algorithm is $O(n^2)$.

Step 4: Scoring.

After the previous processing steps, the similarity between the student’s program and the teacher’s set of programs can be calculated, and the maximum similarity is used to assign a score to the student. The formula for calculating the student’s score is as follows:

14 $$\begin{aligned} \text{ Score } = \text{ PScore } \times {\text {Max}}_{i \in \{0,1, \ldots , n-1\}}\left( {\text {Sim}}_{graph}\left( G_{student}, G_{i}\right) \right) \end{aligned}$$

where PScore is the full score for the question, Sim$_{graph}(G_{student}, G_i)$ refers to the similarity between the student’s PDG and the teacher’s *i*-th PDG, and *n* is the number of programs in the teacher’s set.

## Experiments

### Datasets

To verify the reliability and accuracy of the static program assessment algorithm based on graph semantic similarity, an experimental verification was conducted using two datasets. The first dataset was taken from the final exam of a C language course covering 10 semesters across academic years 2011-2015. Excluding the unanswered questions on programming, 1576 exam papers were retained, with a total of 36 unique programming questions. All questions were manually corrected by teachers. To reduce the scoring errors caused by subjective factors such as fatigue and preference, and to ensure the fairness of the scoring, the project team hired four teachers (all experienced in teaching programming in C) to regrade the test papers one by one. The four teachers provided at least five reference programs for each question, and the full score for each programming question was set to 10 points. When the deviation between two teachers was greater than 2 points, the four teachers discussed and revised the scores together. The teachers were asked to study the reference answers (reference procedures) for the questions carefully before grading.

The second dataset was the program code that passed all the test data in MAXUETANG platform(https://mxt.cn). According to the requirement that only one record for the same subject was retained for the same student, duplicates were deleted. Additionally, subjects with fewer than 50 submission records in the same language were removed and their submission records were deleted. Finally, 856323 submission records and 1370 questions were retained, including 485624 C/C++ submission records, 256283 Java submission records, and 114416 Python submission records. The full score for each question was again set to 10 points. Students’ personal information was filtered in both datasets.

All methods were performed in accordance with the relevant guidelines and regulations of Guizhou Education University. All experimental protocols were approved by the Academic and Ethic Committee at school of mathematics and big data, Guizhou Education University under approval number: GZEU-IRB20230107. The informed consent was obtained from all subjects.

### Assessment indicators

When human teachers grade procedural questions, there will always be scoring errors. The reliability metric is used to analyze the effectiveness of the algorithm scores. If the score assigned by the proposed algorithm is within the given error margin of all teachers’ scores, it is considered an effective score; otherwise, it is an invalid score. Thus, the reliability of the proposed algorithm can be defined as the ratio of the number of valid scores (*M*) to the total number of ratings (*N*), namely:

15 $$\begin{aligned} { Reliability }=\frac{M}{N} \times 100 \% \end{aligned}$$

The precision metric analyzes how close the score assigned by the proposed algorithm is to that assigned by a human teacher. A higher value of the precision indicates that the two are closer together. The precision is calculated as follows:

16 $$\begin{aligned} { Precision }=\left[ 1-\frac{1}{N} \sum _{i=1}^{N}\left( \frac{\left| \overline{x_{i}}-\frac{1}{H} \sum _{j=1}^{H} x_{i j}\right| }{S}\right) \right] \times 100 \% \end{aligned}$$

Where *N* is the number of student programs being assessed, *H* represents the number of human teachers participating in the assessment, *S* is the item score, $\overline{x_{i}}$ represents the proposed algorithm’s assessment score for the *i*-th program, and $x_{ij}$ is the assessment score assigned by the *j*-th human teacher for the *i*th program.

Cosine similarity is used to represent the closeness between algorithm score vector A and teacher score vector B, with a value range of [$-1, 1$]. The closer the value is to 1, the more similar they are. The calculation formula is as follows:

17 $$\begin{aligned} {Cosine\_Similarity} = \frac{\sum _{i=1}^{n} A_i \times B_i}{\sqrt{\sum _{i=1}^{n} A_i^{2}} \times \sqrt{\sum _{i=1}^{n} B_i^{2}}} \end{aligned}$$

Where $A_i$ and $B_i$ represent the *i*-th score from the algorithm and the *i*-th score from the teacher, and *n* represents the total number of scores.

### Experimental results

The algorithm implementation was written in Python and Java, and the experiments were conducted on a hardware environment consisting of an Intel(R) Core(TM) i5-9400 CPU @ 2.90 GHz (6 CPUs), 8G memory, and the CentOS6.10 operating system. The experimental results are shown in Tables 1 and 2.

**Table 1:** Experimental results on dataset 1.

Methods	Average score	Standard deviation	Reliability (%)	Precision (%)	Cosine Similarity
LSA^10^	6.98	2.432	62.11	79.21	0.9232
TF-IDF^12^	6.88	2.473	67.89	80.03	0.9213
STF^13^	6.84	2.239	79.75	87.02	0.9581
PAGCSPF^11^	6.95	2.190	83.62	94.59	0.9839
Ours	7.14	2.173	85.72	96.99	0.9952

**Table 2:** Experimental results on dataset 2.

Methods	Language	Average score	Standard deviation	Reliability (%)	Precision (%)	Cosine similarity
LSA^10^	C/C++	8.09	2.326	60.17	80.92	0.9610
	Java	8.39	2.217	67.28	83.94	0.9668
	Python	8.26	2.231	65.19	82.64	0.9654
	Avg	8.25	2.258	64.215	82.50	0.9644
TF-IDF^12^	C/C++	8.59	2.141	71.23	85.91	0.9703
	Java	8.68	2.079	73.61	86.8	0.9724
	Python	8.63	2.073	73.52	86.31	0.9723
	Avg	8.63	2.098	72.79	86.34	0.9717
STF^13^	C/C++	9.11	1.794	82.63	91.05	0.9811
	Java	9.03	1.844	81.29	90.29	0.9797
	Python	9.13	1.711	84.34	91.30	0.9829
	Avg	9.09	1.783	82.75	90.88	0.9812
PAGCSPF^11^	C/C++	9.14	1.756	83.56	91.48	0.9820
	Java	9.23	1.678	85.43	92.32	0.9838
	Python	9.14	1.701	84.64	91.49	0.9831
	Avg	9.17	1.712	84.54	91.76	0.9830
Ours	C/C++	9.32	1.568	87.59	93.23	0.9861
	Java	9.27	1.596	86.98	92.79	0.9855
	Python	9.15	1.678	85.03	91.51	0.9836
	Avg	9.25	1.614	86.53	92.51	0.9851

**Figure 9:**
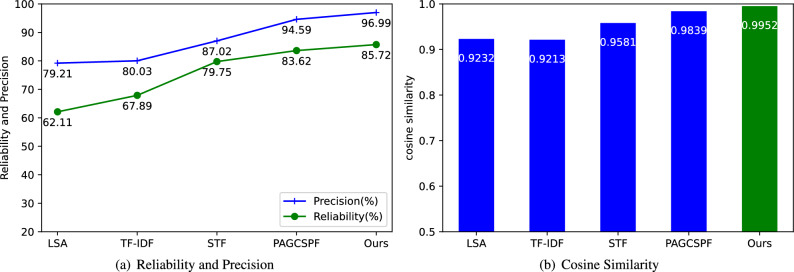
Comparison of algorithm execution results.

**Figure 10:**
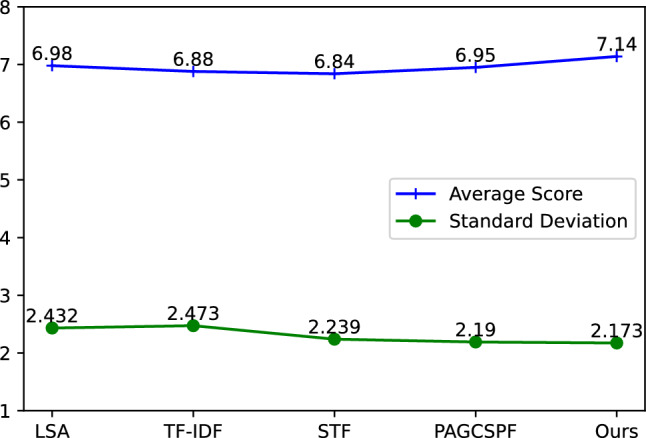
Average score and standard deviation of different algorithms.

On dataset 1, the teacher scoring error was set to 2 points, and the average grade given by the teacher is 7.29. The proposed algorithm was compared with the LAS, TF-IDF, STF, and PAGCSPF algorithms. The experimental results presented in Table 1 show that the proposed algorithm achieves a reliability of 85.72% and a precision of 96.99%. As shown in Fig. 9a, the algorithm proposed in this article has higher reliability and precision than the other four algorithms. These scores are better than those of the four comparison algorithms. The evaluation results are more stable and reliable. Furthermore, as shown in Fig. 9b, the cosine similarity of the algorithm discussed in this paper stands at 0.9952, exceeding that of the other four comparative algorithms. As shown in Fig. 10, the average score of the algorithm proposed in this article is 7.14, which is only 0.15 lower than the average evaluation score of 7.29 given by the teacher, indicating a small difference. In comparison to the other four algorithms, the standard deviation of the proposed algorithm is 2.173, which is lower, indicating higher consistency. This result underscores that the proposed algorithm more closely approximates the teacher’s scoring, exhibits greater effectiveness, and represents a significant improvement over existing methods.

On dataset 2, 40% of the submitted records were randomly selected for each topic as the teacher’s programs, with the remaining 60% used as the student’s programs to be assessed. The scoring error was set to 1 point. The proposed algorithm was again compared with the LAS, TF-IDF, STF, and PAGCSPF algorithms. The experimental results presented in Table 2 show that, in the C/C++ language program assessment, the proposed algorithm achieves a reliability score of 87.59% and precision of 93.23%, outperforming the other four algorithms. In the Java language program assessment, the proposed algorithm again outperforms the other algorithms, with a reliability of 86.98% and precision of 92.79%. In the Python language program assessment, the reliability of the proposed algorithm is 85.03% and its precision is 91.51%, which are better scores than achieved by the other four algorithms. Furthermore, looking at the average scores, the algorithm proposed in this article shows high marks in the evaluations of the three programming languages, implying that it can accurately match the scores expected by teachers. For instance, in the evaluation of C/C++ programs, the algorithm has an average score of 9.32, which is the closest to the actual score, meaning it aligns most closely with the teacher’s grading, and also appears to be more reliable in the credibility of the scores given. Observing the standard deviation, the stability of the algorithm’s scoring is confirmed once again. In the evaluations of the three different programming languages, the algorithm proposed in this article exhibited the lowest standard deviation, thus proving its stable and consistent scoring characteristics. For example, in the evaluation of C/C++ programs, the algorithm’s standard deviation is 1.568, which is lower compared to other algorithms, indicating that it has better robustness and consistency than the other four algorithms compared, giving it an advantage in the field of automated program evaluation. It is evident that the algorithm proposed in this paper has demonstrated excellent performance across various evaluation metrics, particularly when it comes to assessing programs written in different programming languages. Whether in terms of reliability, precision, or consistency of scoring, it shows a significant improvement over the LAS, TF-IDF, STF, and PAGCSPF algorithms. The proposed algorithm can be applied to the evaluation of programs written in multiple languages.

## Conclusion

This paper has proposed an automatic assessment algorithm for programming exercises based on graph semantic similarity. The algorithm calculates the similarity between the students’ programs and the teacher’s programs in terms of the structural similarity of PDGs and the semantic similarity of nodes. Based on the typical reviewing behavior of human teachers, a new optimal similar node path matching algorithm was proposed. This improves the efficiency of calculating the similarity of PDGs, achieving a time complexity of $O(n^2)$. Experiments on two datasets show that the proposed algorithm is superior to LSA, TF-IDF, STF, and PAGCSPF algorithms in terms of reliability and accuracy. The program assessment algorithm proposed in this paper can be used to assess code that cannot be compiled correctly, and supports the assessment of programs written in C/C++, Java, Python, and other languages. The accuracy of the evaluation algorithm proposed in this paper depends, to some extent, on the number of teacher program templates. In future work, we will integrate dynamic evaluation to discover and incorporate innovative student code, supplementing it into the teacher templates. Simultaneously, we will further investigate AI-based techniques for generating teacher program templates to automatically create richer and more diverse templates, thereby enhancing the accuracy of the evaluation algorithm.

## Data Availability

The datasets used and analysed during the current study available from the corresponding author on reasonable request.
